# Breaking the Barrier: The Role of Proinflammatory Cytokines in BBB Dysfunction

**DOI:** 10.3390/ijms26083532

**Published:** 2025-04-09

**Authors:** Małgorzata Gryka-Marton, Anna D. Grabowska, Dariusz Szukiewicz

**Affiliations:** Laboratory of the Blood–Brain Barrier, Department of Biophysics, Physiology & Pathophysiology, Faculty of Health Sciences, Medical University of Warsaw, Chalubinskiego 5, 02-004 Warsaw, Poland; mgrykamarton@gmail.com (M.G.-M.); anna.sepulveda@wum.edu.pl (A.D.G.)

**Keywords:** blood–brain barrier structure, blood–brain barrier integrity, proinflammatory cytokines, neuroinflammation, brain endothelial cells, tight junctions, pericyte–endothelial interaction, astrocytes, neurodegenerative processes

## Abstract

The BBB is created by a special system of brain microvascular endothelial cells (BMECs), pericytes (PCs), the capillary basement membrane, and the terminal branches (“end-feet”) of astrocytes (ACs). The key function of the BBB is to protect the central nervous system (CNS) from potentially harmful/toxic substances in the bloodstream by selectively controlling the entry of cells and molecules, including nutrients and components of the immune system. The loss of BBB integrity in response to neuroinflammation, as manifested by an increase in permeability, depends predominantly on the activity of proinflammatory cytokines. However, the pathomechanism of structural and functional changes in the BBB under the influence of individual cytokines is still poorly understood. This review summarizes the current state of knowledge on this topic, which is important from both pathophysiological and therapeutic points of view. The structures and functions of all components of the BBB are reviewed, with emphasis given to differences between this and other locations of the circulatory system. The protein composition of the interendothelial tight junctions in the context of regulating BBB permeability is presented, as is the role of pericyte–BMEC interactions in the exchange of metabolites, ions, and nucleic acids. Finally, the documented actions of proinflammatory cytokines within the BBB are discussed.

## 1. Introduction

The blood–brain barrier (BBB), a specialized internal barrier between the blood and nervous tissue, plays an important role in ensuring the correct functioning of the central nervous system (CNS), regulating the vital functions of the body as a whole [1]. The BBB complex structure is formed of endothelial cells, pericytes, and astrocytes located between the brain parenchyma and the vascular system and is highly connected with surrounding neurons and microglia [2,3]. A loss of BBB integrity can lead to changes in the composition of the cerebrospinal fluid (CSF) and damage to the CNS. BBB disturbances commonly occur in response to neuronal dysfunction, neuroinflammation, neurodegeneration, and substance use disorders [4,5]. A precise understanding of the structure of the BBB and the pathomechanism by which proinflammatory cytokines influence its disruption may contribute to improvements in existing preventive and therapeutic methods [6,7].

The majority of available literature data consider the disruption of the BBB structure/function in the context of the development of a specific disease (diabetes mellitus, multiple sclerosis, Alzheimer’s disease, neuropsychiatric systemic lupus erythematosus, Herpes simplex encephalitis, and cerebrovascular ischemia), pathological condition, or process (stress, traumatic brain injury, subarachnoid hemorrhage, and sepsis) [8,9,10]. For example, the impact of dysglycemia associated with diabetes mellitus on the BBB structure was reviewed by Watroba et al. [11]. According to the authors, damage to the BBB structure caused by impaired blood sugar stability, especially by the hyperglycemic state (exceeding the normal level), leads to damage to CNS cells, provoking neurodegeneration and dementia. This hypothesis was supported by research studies showing that hyperglycemia increases the expression of proinflammatory cytokines, such as tumor necrosis factor alpha (TNF-α) and interleukins (IL-1, IL-4 and IL-6), even though the detailed mechanism underlying their influence on the integrity and permeability of the BBB has not yet been determined [12]. Małkiewicz et al. [13] reviewed the impact of substance abuse on the BBB, obtaining reliable data on the BBB permeability increase and disruption on one hand and BBB integration preservation on the other hand, due to the activation of certain mechanisms caused by the use of psychoactive substances. Lesniak et al. [14] used a mouse model to investigate how the loss of brain-derived neurotrophic factor due to the BBB disruption caused by congenital or mild traumatic brain injury correlates with depression-like behavior. Although the mechanism of damage to the barrier structure was not described in detail, this study emphasises the possibility of using animal models to study the effects of cytokines on BBB disruption in vivo. In another study, Bronisz et al. [15] assessed the serum levels of molecules associated with changes in BBB structure in patients with epilepsy: matrix metalloproteinases 2 and 9 (MMP-2 and MMP-9), tissue inhibitors of MMP-1 and MMP-2 (TIMP-1 and TIMP-2), the S100 calcium-binding protein beta (S100B), the chemokine CCL-2, intercellular adhesion molecule-1 (ICAM-1), P-selectin, and thrombospondin-2 (TSP-2). The researchers documented that chronic disruption and restoration of the BBB structure are accompanied by increased serum levels of MMP-9, MMP-2, TIMP-1, TIMP-2, and S100B, indicating their direct influence on BBB permeability; however, this study did not provide a detailed explanation of the underlying mechanisms. Łach et al. [16] conducted a comparative analysis of methods in order to identify parameters crucial for BBB activity and to propose an experimental in vitro or in vivo model adequate for studying BBB structure and function. The results of these theoretical and comparative studies shed light on the mechanisms underlying the BBB structure disruption.

While systemic inflammation is a response to pathological changes associated with the development of diseases, some of which are the cause and some of which are the consequence of BBB disruption, specific effects on BBB structure/function may be directly related to the activity of single proinflammatory cytokines [17,18,19]. This review aims to summarize the current state of knowledge concerning the effects of proinflammatory cytokines on the integrity and permeability of the BBB, which are important from both pathophysiological and therapeutic points of view [20,21].

## 2. Methodology of the Literature Search

The investigation into the structure of the blood–brain barrier (BBB) and the impact of proinflammatory cytokines on its disruption was conducted through comprehensive analysis, synthesis of relevant research, and generalization of the collected data. This approach established a coherent framework for presenting findings, supporting a qualitative analysis of the topic and facilitating the achievement of the research objectives.

The analysis focused on understanding the characteristics of the BBB, its structure, and its functions. This approach allowed for the identification of pathways and factors contributing to BBB disruption, mechanisms by which proinflammatory cytokines traverse the BBB without compromising its structure, and the specific effects of these cytokines on BBB integrity. The research materials were selected from highly cited, peer-reviewed publications in the fields of anatomy, neurology, immunology, and microbiology, available through the electronic databases PubMed, Google Scholar, and Scopus.

The literature pertaining to the structure and functions of the BBB was identified using keywords, such as “blood-brain barrier structure”, “capillary endothelium”, “basement membrane”, “astrocytes”, and “blood-brain barrier functions”. Priority was given to studies providing observational or experimental evidence.

Sources exploring proinflammatory cytokines that can cross the BBB without causing structural damage were chosen from research combining theoretical models with experimental findings. Relevant keywords included “proinflammatory cytokines and the blood-brain barrier”, “types of proinflammatory cytokines that cross the blood-brain barrier”, and “ways of crossing the blood-brain barrier by proinflammatory cytokines”.

To examine how proinflammatory cytokines contribute to BBB disruption, a search was conducted using keywords, such as “the role of the inflammatory process in blood-brain barrier integrity disruption”, “negative impact of proinflammatory cytokines on the blood-brain barrier”, and “impact of proinflammatory cytokines on blood-brain barrier structure disruption”.

The collected data were synthesized to elucidate the mechanisms through which proinflammatory cytokines influence BBB disruption. The findings were further summarized to differentiate between the positive and negative effects of proinflammatory cytokines on BBB integrity. Additionally, strategies to mitigate cytokine-induced damage were identified, along with potential applications of BBB permeability in addressing specific medical challenges.

## 3. Normal BBB Structure

The blood–brain barrier (BBB) serves protective and regulatory functions, preventing harmful substances from entering the central nervous system (CNS) [22]. Damage and dysfunction of the BBB compromise the isolation of the brain and nervous tissue from the blood, affecting cerebrospinal fluid stability and neurological health [23,24]. The BBB is present in most of the CNS, apart from circumventricular organs (CVOs), such as the hypothalamus, pineal gland, median eminence, and the vomiting center, which monitor blood composition and regulate autonomic and endocrine functions [25,26].

**Figure 1:**
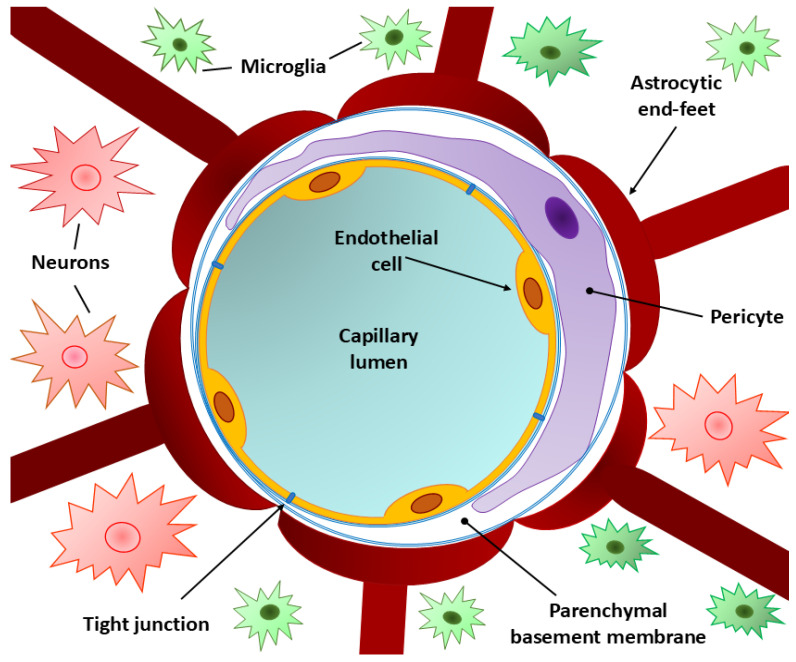
Schematic representation of the blood–brain barrier (BBB) in a cross-section illustrating its cellular counterparts [28,31]. From the capillary lumen, brain microvascular endothelial cells (BMECs) are surrounded by pericytes located within the parenchymal basement membrane. The presence of tight junctions between endothelial cells maintains the integrity and required level of permeability of the BBB. The surface of the basement membrane is covered by the astrocytic end-feet. Microglia and neuronal synapse endings are located in the extracellular matrix surrounding the BBB from the side of the nervous tissue.

The BBB consists of three main structural components: the capillary endothelium, the basement membrane, and the perivascular membrane formed by astrocytic end-feet [1,3,20]. Tight junctions between endothelial cells create a seal that limits solute and water penetration [3,22]. This multi-layered structure provides a sequential defense: the capillary endothelium restricts bacterial and macromolecular entry, the basement membrane maintains barrier integrity and signaling, and the perivascular membrane stabilizes BBB structure [27,28,29,30] (Figure 1).

### 3.1. Endothelial Cells

The capillary endothelium of the blood–brain barrier (BBB) consists of tightly connected endothelial cells, linked by interendothelial tight junctions. Unlike endothelial cells in other body regions, BBB endothelial cells are more flattened, contain more mitochondria, and have fewer caveolae, which selectively reduce capillary permeability [32,33], thus limiting the paracellular transport of hydrophilic molecules while allowing small lipophilic molecules to diffuse. Specific transport mechanisms facilitate nutrient uptake (e.g., glucose via GLUT1) and the passage of oxygen, carbon dioxide, lipids, anesthetics, ethanol, and nicotine. Receptor-mediated endocytosis enables the transport of insulin, transferrin, and leptin across the BBB [34,35,36]. Additionally, the endothelial glycocalyx contributes to BBB homeostasis, regulating the vascular permeability, inflammatory response, and coagulation [37].

### 3.2. Tight and Adherence Junctions

Adherens junctions are intercellular structures that mechanically link neighboring cells through transmembrane proteins called cadherins, maintaining tissue integrity. On the cytoplasmic side, they connect to the actin cytoskeleton via adaptor proteins, predominantly catenins, enabling cellular stability and facilitating intercellular signaling. Cadherins are calcium-dependent adhesion molecules responsible for cell–cell recognition and adhesion. They form homotypic connections with cadherins on adjacent cells, essential for tissue organization. Examples include E-cadherin in epithelial cells, N-cadherin in neural tissues, and P-cadherin in the placenta and epidermis. Catenins, including α-, β-, and γ-catenin, function as cytoplasmic adaptor proteins linking cadherins to the actin cytoskeleton. β-catenin directly binds the intracellular domain of cadherins, associating them with α-catenin, which anchors the complex to actin filaments. Beyond structural roles, β-catenin participates in signaling pathways, notably Wnt signaling, influencing gene expression and cellular differentiation.

**Figure 2:**
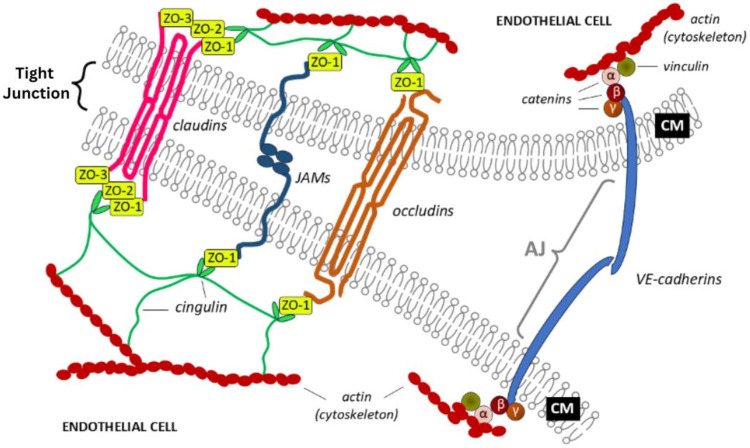
Schematic representation of the tight contact between adjacent endothelial cells [38]. Tight junctions are formed by the joining of major cellular membrane (CM) proteins of adjacent cells, such as claudins, occludins, and junctional adhesion molecules (JAMs). Additional cytoplasmic proteins of adjacent cells participate in tight junction stabilization by binding to elements of the cytoskeleton (actin) of endotheliocytes. These cytoplasmic proteins include zonula occludens-1, -2, and -3 (ZO-1, ZO-2, and ZO-3, respectively); cingulin; vinculin; and catenins (α, β, and γ). A much weaker adherens junction (AJ) connects the adjoining plasma membranes with the electron-dense fibrillary molecules known as vascular endothelial (VE)-cadherins.

Tight junctions are composed of membrane proteins, such as *claudins, occludin, and junctional adhesion molecules (JAMs)*, which form the primary sealing strands. These transmembrane proteins are anchored and regulated by cytoplasmic scaffold proteins, including *zonula occludens-1 (ZO-1), zonula occludens-2 (ZO-2), zonula occludens-3 (ZO-3), cingulin, and vinculin*. The cytoplasmic proteins connect tight junctions to the actin cytoskeleton, providing structural support and facilitating signal transduction necessary for maintaining the selective permeability and integrity of the blood–brain barrier (BBB) [38,39] (Figure 2).

Claudins are integral membrane proteins forming tight junctions (TJs) between endothelial cells, creating a robust barrier through interactions with zonula occludens proteins (ZO-1, ZO-2, ZO-3), and regulating blood–brain barrier (BBB) permeability. Dysfunction or damage to claudins increases monocyte infiltration in inflammatory diseases and is linked with various CNS pathologies, such as brain tumors, stroke, and inflammation [40,41,42,43,44,45]. Occludins, larger proteins that complement claudins, regulate paracellular permeability by working cooperatively within TJs [46,47,48]. Junctional adhesion molecules (JAMs) also strengthen junctional adhesion; however, their precise role in monocyte migration remains unclear [49].

Tight junctions (TJs) and adherens junctions (AJs) collectively maintain BBB integrity and endothelial function. TJs regulate the paracellular movement of ions and solutes, while AJs primarily provide structural stability through cell–cell adhesion. Notably, AJ formation typically precedes and supports TJ assembly, highlighting their functional interdependence.

AJs in endothelial cells predominantly contain vascular endothelial cadherin (VE-cadherin), interacting intracellularly with catenins (β-catenin, p120-catenin). These catenins anchor VE-cadherin to the actin cytoskeleton, strengthening junctional stability. VE-cadherin forms homophilic interactions between adjacent cells, essential for establishing robust cell–cell contacts and facilitating subsequent TJ formation [50,51].

TJs are composed of transmembrane proteins, such as occludin, claudins, and JAMs, linked intracellularly to cytoplasmic scaffolding proteins like ZO-1. ZO-1 serves as a critical anchor to the actin cytoskeleton and facilitates interaction with AJ components, underscoring the complex interplay between TJ and AJ systems. Importantly, VE-cadherin-dependent signaling has been shown to influence TJ protein expression and localization, confirming functional integration between these junction types [52].

Recent research demonstrates that BBB disruption in prion diseases can result directly from proinflammatory cytokines produced by reactive astrocytes, notably IL-6. In prion-infected mice, BBB impairment precedes clinical symptoms, characterized by reduced VE-cadherin expression, compromising endothelial cell cohesion. Reactive astrocytes activated during prion infection release elevated IL-6 levels, significantly impairing junctional integrity. Indeed, endothelial cells from healthy animals exposed to either reactive astrocytes or media conditioned by these cells develop dysfunction comparable to endothelial cells from infected animals. Moreover, direct IL-6 exposure alone reduces trans-endothelial electrical resistance (TEER), indicating compromised barrier integrity. This evidence identifies astrocyte-derived IL-6 as a key mediator of BBB disruption, providing valuable insight into early pathogenesis in prion diseases and highlighting astrocytes and their cytokine products as promising therapeutic targets [53].

### 3.3. Pericytes

Pericytes are located outside the capillary endothelium and share with it the basement membrane. They regulate BBB permeability, support endothelial cells, and neutralize toxic metabolites [54,55,56,57,58]. By expression of vasopressin, angiotensin, endothelin receptors, and BBB-specific genes, they play a role in cerebral autoregulation and in the induction of the polarization of astrocyte processes that envelop the CNS capillaries [59,60]. Pericyte dysfunction is linked to neurodegenerative diseases, such as Alzheimer’s and multiple sclerosis [54,55,56,57,58,59,60,61,62,63,64,65,66,67]. Identification of species-specific pericyte subtypes (differing between humans and animals) highlights the need for their precise characterisation, serving new therapeutic approaches that specifically target disease-associated pericyte subtypes in individuals with neurodegenerative diseases [68,69].

### 3.4. Basement Membrane

The basement membrane of capillaries is an extracellular matrix located under endothelial cells, supporting BBB structure and facilitating intercellular signaling [70]. It consists of collagen IV, laminin, nidogen, and perlecan synthesized by endothelial cells, pericytes, and astrocytes [70,71]. Most abundant collagen IV maintains membrane stability and vascular integrity. Laminin, particularly regarding astrocyte-derived isoforms, is essential for BBB maintenance. Nidogen (entactin) stabilizes collagen IV and laminin and perlecan contributes to the basement membrane formation, yet the role of the latter two proteins in BBB integrity remains unclear [28,72,73,74,75,76,77,78,79].

### 3.5. Perivascular Membrane

The perivascular membrane, formed by astrocytic processes (“feet”), plays a role in BBB maintenance and its dysfunction may lead to neuroinflammation. Astrocytes, though not involved in initial BBB formation, contribute to its stability by modulating tight junction proteins through vascular endothelial growth factor (VEGF) and angiotensin-1 expression [80,81,82,83].

Understanding the structure, function, and interaction of all the above BBB components is crucial for assessing the impact of proinflammatory cytokines on barrier integrity.

## 4. Effects of Proinflammatory Cytokines on the BBB Structure and Integrity

Cytokines are a broad category of small, soluble proteins secreted by various cells, primarily immune cells, that play a crucial role in regulating immunity, inflammation, and hematopoiesis. They serve as fundamental mediators of cell-to-cell communication, enabling the coordination of immune responses and the maintenance of homeostasis.

Several subgroups of cytokines are distinguished based on their structural characteristics and functions. Interleukins (ILs) are proteins mainly produced by leukocytes that regulate immune and inflammatory responses. Tumor necrosis factors (TNFs) are a group of cytokines involved in systemic inflammation and the activation of immune cells. Interferons (IFNs) play a key role in antiviral defense and the modulation of immune responses. An important subset of cytokines is chemokines, characterized by their ability to induce chemotaxis, guiding the directed migration of immune cells to sites of inflammation or injury.

In the context of the blood–brain barrier (BBB), proinflammatory cytokines, such as IL-1β, IL-6, TNF-α, and IFN-γ, are well-known for their disruptive effects on barrier integrity through various mechanisms. However, chemokines also play a significant role in modulating BBB permeability by influencing the migration of immune cells across the barrier. To provide a comprehensive overview of cytokine impact on BBB structure and function, this paper addresses not only classical cytokines, such as TNF-α, IL-1β, and IL-6, but also chemokines, whose role in this context is equally important.

**Figure 3:**
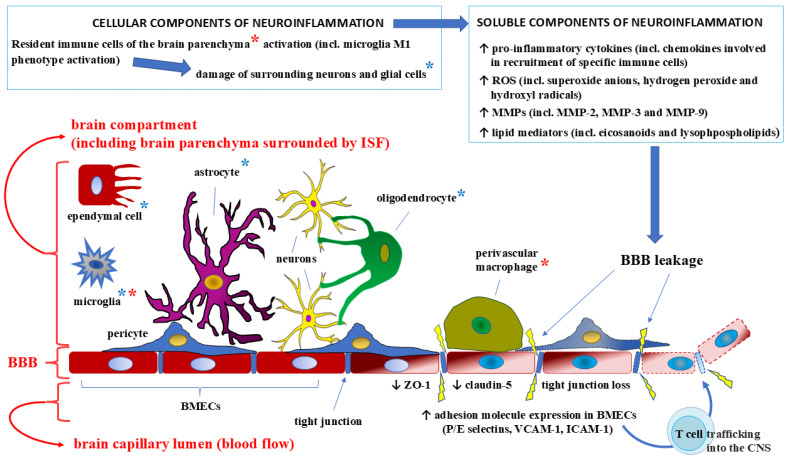
The endogenous pathway of proinflammatory cytokines through the blood–brain barrier (BBB) [11,17]. Neuroinflammation may lead to decreased BBB integrity due to the activation of brain-resident immune cells (marked with red asterisks), which then results in the damage of surrounding neurons and glial cells (marked with blue asterisks). The cellular components of neuroinflammation (summarized in the box on the left) are accompanied by soluble components, such as overproduced proinflammatory cytokines and other inflammatory mediators (listed in the box on the right) in inflammation-activated cells (e.g., in polarized states towards a proinflammatory (M1) phenotype microglia). Decreased expression of membrane proteins (e.g., claudin-5) and cytoplasmic proteins (e.g., zonula occludens-1 (ZO-1)) is accompanied by the loosening of intercellular tight junctions. Combined with an increase in the expression of adhesion molecules on the surface of the brain microvascular endothelial cells (BMECs), an increase in BBB permeability may result in the influx of T cells from the bloodstream into the central nervous system (CNS). Other abbreviations: ROS—reactive oxygen species, MMPs—matrix metalloproteases.

The effects of cytokines on the BBB structure depend on the mechanism of barrier penetration [84]. When exogenous cytokines enter the body, for example, through injection, they can quickly and easily cross the BBB without disrupting its structure. This process can occur through retrograde axonal transport, saturated influx transport (SIT), or simple diffusion in areas of the brain where the BBB is incomplete. Retrograde axonal transport refers to the directed movement of cytokine molecules along microtubules from the distal neuron regions/periphery toward the cell body (soma) while SIT denotes a state in which some influx transporters can become saturated at high cytokine concentrations, leading to a plateau in uptake rates, described by Michaelis–Menten kinetics. The areas of facilitated diffusion correspond to those described previously, CVOs. The cytokines IL-1α, IL-6, and TNF-α can cross the barrier via CVO-related pathways [85,86]. Usually, the route of exogenous cytokine transport into the body is controlled and, thus, it does not significantly disturb its structure. However, this route of penetration can compromise the integrity of the BBB by activating cytoplasmic free calcium and potentially disrupting its homeostasis in the brain [87]. Calcium (Ca^2+^), released from cell organelles into the cytoplasm, is a key intracellular messenger in many cell types, including BMECs [88], considered to be the trigger for secretory exocytosis [89]. The BBB penetration of endogenous proinflammatory cytokines can be more dangerous for the structural integrity of the barrier. Damage to the BBB structure and, accordingly, an increase in its permeability are most often associated with various neurological disorders, psychiatric disorders, and neurodegenerative and infectious diseases of the CNS, which increase the levels of proinflammatory cytokines [18,90] (Figure 3).

The higher probability of BBB structural changes related to the endogenous pathway of CK BBB penetration may be related to the duration of sustained elevated levels of proinflammatory cytokines, which is usually chronic in the above-mentioned CNS diseases. It was suggested that during the course of CNS diseases, the expression of an important membrane protein, the major facilitator superfamily domain containing 2a (MFSD2A), on BMECs is reduced. MFSD2A expression is regulated by pericytes, which thus influence transcytosis, the phenomenon of transporting a given substance from one pole of the cell to the other through the cytoplasm.

Therefore, the reduced MFSD2A occurring during CNS diseases or injuries can increase the levels of transcellular vesicles, including caveolin-1 (CAV-1), nuclear factor erythroid 2 p45-related factor 2 (NRF-2), and heme oxygenase 1 (HO-1), in BMECs, which may contain toxins, pathogens, or cytokines in brain, while the upregulation of MFSD2A will protect BBB from decreasing levels of the vesicles [90].

In response to pathological conditions within the CNS, the immune system uses inflammation as one of its means to resolve the pathological process [91]. The inflammatory response is mobilized by proinflammatory cytokines produced by macrophages, including brain-resident microglia, as well as leukocytes, neutrophils, astrocytes, or other cells, depending on the pathological process [92]. For example, in response to a stroke or lipopolysaccharide, the proinflammatory cytokines IL-1 and TNF-α are produced by microglia, which are activated in a proinflammatory phenotype during neuroinflammation to induce the formation of reactive astrocytes that acquire the same proinflammatory phenotype [93]. The effects of proinflammatory cytokines, chemokines, and histamine on BBB disorders are summarised in Table 1.

Depending on the pathophysiology of inflammation, the profiles and concentrations of proinflammatory cytokines may show significant differences in specific diseases of the CNS [141]. Autoimmune diseases indirectly affect the permeability of the BBB through the entry of autoreactive T lymphocytes into the CNS, releasing there the proinflammatory cytokines IL-1, TNF-α, and interferon γ. In the development of multiple sclerosis, this triggers the process of an autoimmune attack on the myelin sheath [142,143]. The CNS release of cytokines, along with oxidants and proteolytic enzymes, also occurs in ischemic stroke. The ingress of these elements into the brain tissue provokes the development of cytotoxic edema and increases the BBB permeability, which leads to the penetration of leukocytes through the capillary endothelium into the brain tissue and to the neuronal damage [144]. In the pathogenesis of epilepsy, despite the experts’ disagreement on the primary pathological process (seizures or damage to the BBB), the main neuroinflammatory process is considered to be the mobilization of the cytokines IL-1β, IL-6, and TNF-α in response to pathological changes in the CNS caused by traumatic brain injury (TBI), brain tumors, stroke, drug exposure, etc. [145,146]. Agreeing on TBI as one of the most common causes of epilepsy opens investigations on the association of BBB damage with seizures. Depending on the consequences of TBI, BBB disruption occurs mechanically or as a result of the activity of proinflammatory cytokines released and/or activated in response to trauma. Blood components, one of which is thrombin, enter the CSF through a hemorrhage, where they provoke neuronal excitability and, accordingly, seizures [147,148]. One of the proteins playing an important role in the inflammatory response to TBI and affecting BBB integrity is the neuronal tau (*τ*) protein that supports axonal transport and microtubule dynamics [149]. Tau preserves cellular integrity and shifts cellular pathways away from pro-cell-death signaling cascades. Tauopathies represent a heterogeneous group of approximately 20 different neurodegenerative diseases characterized by the abnormal deposition of the microtubule-associated protein tau (MAPT) in cells of the nervous system [150]. The BBB damage observed in individuals with tauopathies without amyloid-β overproduction suggests a role for tau in BBB damage that is driven by chronic neuroinflammation initiated within the microglial compartment by extracellular tau aggregates (e-*τ*) released from injured neurons [151]. The increase in the levels of proinflammatory cytokines (IL-1β, IL-6, IL-12, TNF-α, and IFN-γ) is also influenced by several mental disorders, including depression. Inflammatory activity can both provoke and maintain a specific (proinflammatory) cytokine profile and so prolonged exposure of the CNS to this state alters BBB permeability and, accordingly, provokes other neurological disorders and diseases [152]. The activation of astrocytes and increased levels of the cytokines IL-1β, IL-6, and TNF-α have been observed under the influence of neurotoxic drugs. Thus, disruption and increased permeability of the BBB caused by methamphetamine neurotoxicity are manifested by decreased levels of claudin and occludin, the swelling of astrocytes and their processes, a decrease in pericyte coverage, and the loss of tight junctions of endothelial cells [153,154,155]. The factors that provoke increased production of proinflammatory cytokines (IL-6 and TNF-α) include the histamine H4 receptor, which indirectly activates mast cells, and, along with cytokines, actively produces histamine and chemokines [156]. The chemotactic activity of the latter is of key importance in the physiology and pathophysiology of the CNS [133,157,158,159].

Under physiological conditions in the nervous system, chemokines contribute to cellular interactions, the activation of signaling pathways, and the maintenance of CNS homeostasis. For example, the chemokines CCL2, CCL19, CCL20, CCL21, and CXCL12 are involved in immunological surveillance by transmitting signals and activating immune cells; CX3CL1 (fractalkine) is involved in maintaining adult neurogenesis, ensuring the balance of homeostasis, and limits the expression of the proinflammatory cytokines IL-1β, IL-6, and TNF-α [134,135]. The chemokines CXCL12, CCL19, CCL20, and CCL21 are expressed in the vascular system of the BBB [132].

Under pathological conditions, chemokines act as mediators of cell migration and participate in the regulation of inflammatory and autoimmune processes in the CNS (differentiation and growth of cells, including tumors) [160]. The mechanism by which CX3CL1 affects BBB integrity depends on the cells involved in migration. For example, one mechanism is triggered by a significant accumulation of CD16+ monocytes on inflamed cerebral endothelial cells due to their transendothelial migration in response to CX3CL1 expression [136]. In addition, the migration of CD4+ T cells is ensured by the chemoattractant activity of CX3CL1 [137]. Changes in BBB permeability are affected by an increase in CXCL13 levels, which occurs as a result of the development of tumors with cerebral metastases. This process leads to an increase in the paracellular permeability of the capillary endothelium and a decrease in the expression and localization of the tight junction proteins claudin-5 and occludin [161].

Histamine, a biogenic amine formed by the conversion of histidine via histidine decarboxylase (HDC), is another molecule affecting the BBB integrity [138,139]. The histamine molecule is too large to penetrate the capillary endothelium and, thus, it cannot cross the BBB unhindered [140]. However, histamine H1 and H2 receptors are expressed on both luminal (blood-facing) and abluminal (brain-facing) plasma membranes of the brain endothelium and regulate several endothelial functions, such as blood coagulation and BBB permeability [162,163]. Histamine is able to increase BBB permeability by downregulating the major tight junction membrane proteins claudin-5 and occludin and the additional cytoplasmic protein ZO-1. The decreased expression of these key proteins is a result of the histamine-induced inhibition of the intracellular PI3K/AKT/mTOR signaling pathway regulating the cell cycle [164]. Moreover, the expression of the histamine H2 receptor can be used as a predictor of the barrier permeability provoked by histamine [164]. Conversely, endothelial histamine H1 receptor signaling reduces BBB permeability and the susceptibility to autoimmune encephalomyelitis [165]. While the detailed mechanism of this response remains to be elucidated, it was shown that reduced BBB permeability following H1R stimulation was accompanied by increases in both intracellular Ca^2+^ and cAMP [165]. Brain tissue should also be considered a separate source of histamine, the abundance of which is determined by the availability of L-histidine and the local activity of HDCs [166]. Newly synthesized neuronal histamine is thought to be stored within nerve terminal vesicles. Both in vivo and in vitro studies have shown that the depolarization of nerve terminals activates the exocytotic release of histamine via a voltage- and calcium-dependent mechanism [167].

## 5. Proinflammatory Cytokines Within the BBB: Clinical Approaches and Potential Therapies

An investigation of the structure of the BBB and the effects of proinflammatory cytokines on damage to the BBB revealed that the cytokines IL-1, IL-6, and TNF-α are most often involved in barrier disruption [132]. Therefore, counteracting the mechanism underlying their effect on the BBB should be addressed in the development of therapeutic approaches to preserve BBB integrity and prevent neurological disorders provoked by the ingress of blood elements into nervous tissue. Moreover, the ability of proinflammatory cytokines to increase BBB permeability can also be used to develop new therapeutic strategies, for example, creating anti-cancer drugs based on cytokine derivatives with good CNS penetration [168,169,170].

Under physiological conditions, all body systems function and interact safely with each other, supporting the process of vital activity. Initiation of the pathological process in the body induces an immune response and its regulators, while performing their tasks at the highest level of intensity, can cause damage to other systems and structures [171,172]. Studies of the BBB revealed that its main functions (protection and regulation) are ensured primarily by the specific features of all the components, from the specific shape and tight connections between endothelial cells to the placement of astrocyte processes in a tight ring around the basement membrane. In response to pathological processes occurring in the nervous system, including autoimmune diseases (multiple sclerosis, Guillain–Barré syndrome, and myasthenia gravis), the immune response involves systemic inflammation induced by proinflammatory cytokines [173,174,175,176,177,178]. As reported in previous studies, a significant increase in the concentration of these cytokines in the bloodstream can lead to BBB damage and increased permeability through a disruption of the tight junctions of capillary endothelial cells and other barrier components [176,177,179,180,181,182]. The experimental data can be used for the development of therapeutic methods to control the levels of proinflammatory cytokines to preserve the integrity of the BBB.

This issue was addressed by Takeshita et al. [183], who built static BBB models to study long-term barrier function and have shown that the IL-6 blockade suppresses BBB disorders, preventing the onset of visual spectrum neuromyelitis. This study revealed that the inhibition of T cell migration to the nervous tissue of the spinal cord was influenced by the blockade of IL-6 signal transduction, which prevented the increase in BBB permeability. IL-6 blockade was achieved by transferring neuromyelitis optica immunoglobulin G (NMO-IgG) autoantibodies and satralizumab through the BBB using a triple culture system, which mimics the close contact of endothelial cells, pericytes, and astrocyte processes [183]. The conclusions of this study are convincing and the obtained results can serve as an example for the development of methods to block other proinflammatory cytokines, such as IL-1β and TNF-α, using analogical BBB models.

As already mentioned, the ability of proinflammatory cytokines to penetrate the BBB should be considered not only to find methods to limit it but also to use it as a tool to overcome the BBB as an obstacle to drug penetration into the nervous tissue. Corti et al. [184] investigated the use of TNF-α as a “conductor” of drugs to cross the BBB to deliver anticancer therapy directly to brain tumors. This team of researchers has studied the capabilities and properties of the drug NGR-TNF, a fusion of the peptide Cys-Asn-Gly-Arg-Cys-Gly (CNGRCG, denoted as NGR) and the cytokine TNF-α, and the ligand of aminopeptidase N (CD13) of tumor-blood-vessel-positive (+) blood vessels. The results of preclinical and clinical trials have shown the effectiveness of this drug in changing the selectivity of the tumor BBB, which has improved the quality of chemotherapy and increased patient survival. However, the authors also noted the problems that could arise due to the instability and molecular heterogeneity of this drug. Given the therapeutic potential of drugs based on the combination of peptides and cytokines, the strategy of this nonimmunogenic approach for the safe treatment of the BBB at the tumor site seems promising, increasing the therapeutic index of cytokines in cancer therapy.

In addition to factors related to exogenous BBB disruption (brain injuries, infections, radiation, etc.), pathological processes within the nervous system provoked by internal causes, such as stress or sleep loss, also affect BBB integrity. The regulatory effect of sleep on the BBB was studied by Hurtado-Alvarado et al. [185]. They reported that sleep deprivation provoked a low-grade inflammatory state. According to the study, increased BBB permeability was observed in experimental mice with limited sleep durations. The hippocampus of these mice presented increased expression of neuroinflammatory markers, such as TNF, IFN-γ, and the adenosine receptor. Notably, BBB disruptions appeared after ten days of prolonged sleep loss or disruption and the damaged BBB structure showed a limited ability to rebuild/heal, which could provoke complex nervous disorders and neurodegenerative diseases.

The pandemic of the acute respiratory disease COVID-19 aroused the interest of researchers in terms of the effects of the highly transmissible, pathogenic severe acute respiratory syndrome coronavirus-2 (SARS-CoV-2) on various body systems. Zhang et al. [186] reported that SARS-CoV-2 penetrates the BBB, which is associated with the destruction of the basement membrane without affecting tight junctions. An in vivo experiment detected the SARS-CoV-2 RNA in the vascular wall, perivascular space, and microvascular endothelial cells of the brains of infected mice and BBB breakdown was observed in infected hamsters. The peculiarity of BBB damage in animals is associated with the fact that the expression of the main proteins forming the tight junctions of endothelial cells (claudin, occludin, and JAMs) does not change but the basement membrane is disrupted. This study reveals the transcellular pathway of BBB damage caused by the SARS-CoV-2 virus, which bypasses tight junctions and breaks the barrier of endothelial cells. In addition, this effect of SARS-CoV-2 on the BBB is dangerous for the integrity of the barrier since the virus damages one of its structural units and activates the immune response in the form of the mobilization of proinflammatory cytokines; moreover, the subsequent recovery process may be significantly complicated or even impossible. However, verification of this statement requires additional research. The SARS-CoV-2 infection is also considered to cause elevated levels of proinflammatory cytokines, referred to as the cytokine storm [187]. When investigating the pathogenesis of the cytokine storm, Soy et al. [188] analysed the experimental data on the topic and reported that the manifestation of such a strong and dangerous process may be associated with the genetic features of SARS-CoV-2, as well as gene mutations in the human body. The consequences of the cytokine storm include a high mortality rate and treatment, such as antiviral, anti-inflammatory, and antirheumatic therapy, is administered to target several processes at once. The effects of cytokine storms on the BBB are poorly understood [189]. However, it is considered that the actions of individual cytokines are additive or even synergistic in relation to the known mechanisms of BBB destruction. For example, TNF-α disrupts the tight junctions of the human brain-like endothelial cell (hBLEC) monolayer and promotes the translocation of claudin-5 to the late endosomes to be subsequently degraded by the lysosomal system [190]. It was also reported that an increase in TNF-α concentration increases BBB permeability by affecting the histone methyltransferase enhancer of zeste homolog 2 (EZH2)–claudin-5 axis [191]. Under the influence of increased TNF-alpha concentrations, occludin is also degraded via the hypoxia-inducible factor 1 (HIF 1α)/VEGF/VEGFR 2/ERK signaling pathway and/or transient stimulation of the p38MAPK and ERK1/2 pathways [192]. Not without significance for the loss of BBB integrity is the fact that under the influence of TNF- α, astrocytes reproduce a canonical event associated with reactive astrogliosis in a time-dependent manner with NF-kB p65 subunit nuclear translocation and a subsequent increase in the gene expression of IL-1β, IL-6, and TNF-α [193]. Consequently, the upregulation of IL-1β suppresses astrocytic signaling protein sonic hedgehog (SHH) production, leading to the downregulation of tight junction proteins in ECs and disintegration of the BBB. Moreover, IL-1β-stimulated astrocytes secrete the proinflammatory chemokines CXCL2, CCL2, and CCL20, which induce the migration of immune cells (e.g., neutrophils, monocytes, dendritic cells, and pathogenic Th cells), thereby worsening BBB disruption and neuroinflammation [97]. Excessive IL-6, in turn, decreases endothelial Wnt/β-catenin signaling through activating the NF-κB pathway, resulting in reduced proliferation and self-renewal of BMECs, and thus promotes acute BBB disruption [183,194].

Researchers have concluded that the mechanism underlying the effect of cytokine storm on the BBB is additive and related to the specific cytokines affected but no studies have determined the level of BBB recovery after successful treatment of the cytokine storm.

### 5.1. Interleukin–6

Interleukin-6 (IL-6) is a key proinflammatory cytokine that plays a pivotal role in blood–brain barrier (BBB) integrity and neuroinflammation. Under normal physiological conditions, it contributes to neuronal survival, neurogenesis, and synaptic plasticity; however, its chronic overexpression disrupts BBB function and contributes to the pathogenesis of various neuroimmune and neurodegenerative disorders. One of the primary mechanisms through which IL-6 affects BBB permeability is by altering the expression of tight junction proteins in endothelial cells, leading to increased paracellular permeability and the infiltration of immune cells into the central nervous system (CNS) [195,196]. Elevated IL-6 levels have been implicated in conditions such as neuromyelitis optica spectrum disorder (NMOSD), multiple sclerosis (MS), Alzheimer’s disease, and neuropsychiatric lupus erythematosus (NPSLE), where it contributes to disease progression by enhancing immune cell trafficking into the CNS and promoting neuroinflammatory damage [197,198,199].

### 5.2. Tumor Necrosis Factor-Alpha (TNF-α)

Tumor necrosis factor-alpha (TNF-α) is a proinflammatory cytokine from the TNF superfamily, critical in immune regulation and inflammatory responses. Produced primarily by macrophages, T cells, and NK cells, TNF-α activates NF-κB and MAPK pathways, promoting cytokine production, leukocyte recruitment, and apoptosis. TNF-α has been shown to act synergistically with IL-6, further exacerbating BBB permeability and allowing for the infiltration of autoreactive lymphocytes and monocytes into the CNS [200]. Both IL-6 and TNF-α disrupt tight junctions by modulating the expression of proteins such as occludin and claudin-5, leading to endothelial barrier breakdown [201]. This cytokine interplay is particularly relevant in autoimmune neuroinflammatory disorders, where sustained activation of IL-6 and TNF-α perpetuates a cycle of chronic inflammation and BBB leakage, ultimately leading to neuronal damage and cognitive impairment [202].

Given its central role in BBB dysfunction and neuroinflammation, IL-6 has emerged as an attractive therapeutic target. Monoclonal antibodies, such as tocilizumab and sartralizumab, have demonstrated efficacy in stabilizing BBB integrity, reducing inflammation-driven neurological damage, and preventing immune cell infiltration into the CNS [203,204]. Tocilizumab, a humanized monoclonal antibody targeting IL-6R, is FDA-approved for the treatment of NMOSD, rheumatoid arthritis, juvenile idiopathic arthritis, and cytokine release syndrome [183,205]. Similarly, sartralizumab, another humanized monoclonal antibody against IL-6R, has received FDA approval for NMOSD treatment, particularly in patients with anti-AQP4 antibodies [204]. These therapies work by blocking IL-6 receptor (IL-6R) signaling, thereby mitigating the downstream inflammatory cascade that exacerbates BBB permeability and neuroinflammatory injury. The effectiveness of the IL-6 blockade has been particularly evident in NMOSD, where IL-6 inhibition reduces disease relapse rates and preserves neurological function [183].

Additionally, TNF-α is a key mediator of BBB dysfunction, neuroinflammation, and reactive astrocytosis. Studies have demonstrated that TNF-α contributes to stress-induced BBB disruption and neuroinflammatory processes through multiple signaling pathways, including the EZH2-claudin-5 axis and TNF-STAT3 axis in reactive astrocytes. Sun et al. (2024) identified that TNF-α promotes BBB permeability by epigenetically repressing claudin-5 expression via EZH2, facilitating peripheral TNF-α infiltration into the hippocampus and exacerbating neuroinflammation linked to depressive symptoms [191]. The pharmacological inhibition of EZH2 or upregulation of claudin-5 restored BBB integrity and reduced TNF-α levels in the brain, mitigating depression-like behaviors.

Kim et al. [206] described a TNF-STAT3 signaling axis in reactive astrocytes as a driver of BBB dysfunction. Using a human-induced pluripotent stem-cell-derived BBB co-culture model, the study showed that TNF-α induces astrocyte transition to an inflammatory state, disrupting the BBB through STAT3 activation and the upregulation of SERPINA3 (α1ACT). This process exacerbates neurovascular inflammation and promotes immune cell infiltration into the CNS. Inhibition of Serpina3n in murine brain cultures reduced TNF-induced BBB disruption, confirming its role in TNF-α-mediated neuroinflammation. Further, intracerebroventricular (ICV) administration of Serpina3n in mice directly induced BBB dysfunction, increased VCAM-1 expression, and decreased claudin-5 levels, reinforcing its significance in neurovascular pathology.

Collectively, these studies illustrate the role of TNF-α in perpetuating BBB dysfunction and neuroinflammation through interconnected pathways, including EZH2-mediated claudin-5 repression and TNF-STAT3-α1ACT signaling in astrocytes. Targeting TNF-α, EZH2, or SERPINA3/α1ACT may represent viable therapeutic strategies for neuroinflammatory disorders. Future research should focus on exploring the therapeutic potential of IL-6 and TNF-α blockades in neurodegenerative and autoimmune conditions characterized by chronic inflammation and BBB impairment. Understanding the intricate cytokine network involved in BBB dysregulation is crucial for developing effective treatments aimed at preserving BBB integrity, reducing neuroinflammation, and improving clinical outcomes in disorders, such as Alzheimer’s disease, multiple sclerosis, and major depressive disorder.

### 5.3. Interleukin-17A

Another cytokine considered critical in causing BBB dysfunction and neuroinflammation is interleukin-17A (IL-17A). Elevated levels of interleukin-17 (IL-17) have been implicated in the disruption of the blood–brain barrier (BBB) across various neurological conditions. IL-17 contributes to BBB breakdown through multiple mechanisms, including endothelial cell contraction, neutrophil recruitment, and the disruption of tight junctions. Studies have shown that IL-17 induces the production of reactive oxygen species (ROS) via NADPH oxidase and xanthine oxidase pathways, leading to oxidative stress that activates the endothelial contractile machinery, ultimately increasing BBB permeability [112]. Additionally, IL-17 promotes the expression of chemokines and adhesion molecules on endothelial cells, thereby facilitating the recruitment and infiltration of neutrophils into the central nervous system (CNS) [6]. This infiltration exacerbates neuroinflammation and further compromises BBB integrity.

Importantly, IL-17 has been found to downregulate the expression of critical tight junction proteins, such as occludin, which are essential for maintaining BBB integrity [112]. The reduction in occludin levels weakens the tight junctions between endothelial cells, increasing BBB permeability and allowing the passage of potentially harmful substances into the CNS. The combination of endothelial contraction, immune cell recruitment, and impairment of tight junction proteins highlights the multifaceted role of IL-17 in promoting BBB dysfunction. Such mechanisms underscore the pathological role of IL-17 in compromising BBB integrity and suggest its potential as a therapeutic target in neuroinflammatory diseases.

Chen et al. [207] demonstrated that nitroglycerin (NTG)-induced chronic migraine in a rat model was associated with increased BBB permeability, allowing IL-17A to cross into the medulla oblongata and activate the trigeminal nucleus caudalis (TNC) through microglia-mediated neuroinflammation [207]. The findings revealed that NTG administration disrupted both functional and structural components of the BBB, altering the expression of key transport proteins, such as the receptor for advanced glycation end products (RAGE), low-density lipoprotein receptor-related protein 1 (LRP1), aquaporin-4 (AQP4), and major facilitator superfamily domain-containing protein 2A (MFSD2A), along with tight junction proteins like zonula occludens-1 (ZO-1), occludin, and VE-cadherin-2 [20]. This increase in BBB permeability facilitated the entry of IL-17A from the periphery into the CNS, triggering neuroinflammation and sensitizing the trigeminal system, a hallmark of migraine pathophysiology. Furthermore, their study demonstrated that IL-17A signaling promoted the release of additional inflammatory cytokines, such as tumor necrosis factor-alpha (TNF-α), interleukin-6 (IL-6), and interleukin-1β (IL-1β), which further exacerbated BBB dysfunction and neuroinflammatory cascades [195]. Interestingly, the inhibition of neuroinflammation using ibuprofen effectively mitigated IL-17A-induced sensitization, suggesting that an IL-17A blockade may be a promising therapeutic target for migraine prevention [207]. These findings highlight the intricate interplay between IL-17A, BBB integrity, and neuroinflammation in chronic migraines and suggest that targeting cytokine-mediated BBB disruption could offer novel therapeutic strategies for neuroimmune disorders beyond migraines, including multiple sclerosis and neuropsychiatric lupus erythematosus.

### 5.4. Interleukin–4

Interleukin-4 (IL-4) is a pleiotropic cytokine primarily recognized for its anti-inflammatory properties within the central nervous system (CNS). It modulates immune responses by promoting the differentiation of naïve T cells into T helper type 2 (Th2) cells and by inducing alternative activation of microglia and macrophages, which enhances their reparative functions and reduces the production of proinflammatory mediators. This modulation contributes to maintaining the integrity of the blood–brain barrier (BBB) under physiological conditions. However, dysregulated IL-4 expression can adversely affect the BBB. For instance, elevated IL-4 levels have been implicated in increased BBB permeability following neonatal hepatitis B vaccination, potentially leading to neurobehavioral impairments [130].

Furthermore, IL-4 interacts with other cytokines, such as tumor necrosis factor-alpha (TNF-α) and interleukin-6 (IL-6), to modulate inflammatory responses. While IL-4 generally suppresses the production of these proinflammatory cytokines, an imbalance can exacerbate neuroinflammation and compromise BBB integrity. Therefore, understanding the nuanced role of IL-4 in BBB regulation is crucial for developing targeted therapies aimed at preserving neurological function and mitigating CNS pathologies.

In terms of its main functions, the BBB plays a pivotal role not only in ensuring the CNS homeostasis but also in the vital activities of the whole organism. Knowledge regarding the effects of proinflammatory cytokines on the BBB structure reviewed in this paper may be useful in the development and implementation of specific therapeutic strategies that, depending on the case, are based on a blockade of some of these cytokines that is safe for the immune system or quickly and effectively restores the integrity of the damaged BBB structure [81].

## 6. Concluding Remarks

This review summarizes the current knowledge of the blood–brain barrier (BBB) structure and the detrimental effects of proinflammatory cytokines. A comprehensive literature review was conducted, focusing on the key components of the BBB and the mechanisms through which proinflammatory cytokines compromise its integrity.

The most crucial structural element of the BBB is the capillary endothelium. Its unique characteristics arise from the specialized arrangement of endothelial cells, reinforced by tight junctions that ensure selective permeability. This selectivity is maintained through the formation of tight junctions involving cytoplasmic proteins (zonula occludens-1, -2, -3; cingulin; α-catenin; β-catenin; γ-catenin; and vinculin) and primary membrane proteins (claudin, occludin, JAMs, and cadherins), supported by the cytoskeletal protein actin.

Pericytes play a vital role in regulating the permeability of interendothelial junctions to fluids and facilitating the exchange of metabolites, RNA, and ions with endothelial cells. The basement membrane, composed of collagen IV, laminin, nidogen, and perlecan, provides structural support, enabling capillary endothelial cells and pericytes to adhere to it, ensuring efficient signal transmission and BBB integrity. Additionally, astrocytes form perivascular membranes through their processes, which further contribute to barrier stability.

The impact of proinflammatory cytokines on BBB structure and permeability is closely related to their primary role in initiating inflammatory responses to pathological processes within the nervous system. Cytokines, such as IL-1β, IL-6, and TNF-α, are frequently implicated in these disruptions. Their interactions with the structural components of the BBB involve the degradation of claudin, reduction of β-catenin levels, and astrocyte damage, ultimately compromising tight junction integrity.

We have expanded our discussion to explicitly address the translational gaps and limitations of current therapeutic approaches targeting cytokine-mediated BBB disruption. Although preclinical studies using cytokine inhibitors, such as TNF-α and IL-6 blockers, have demonstrated promising results in protecting the BBB in animal models, these successes have not consistently translated to clinical practice. Several critical challenges contribute to this discrepancy.

First, the redundancy and complexity of cytokine pathways often result in limited efficacy when blocking a single cytokine. This redundancy can lead to compensatory mechanisms that undermine therapeutic interventions. Second, systemic cytokine inhibition carries the risk of significant side effects, including immunosuppression and altered signaling in non-target tissues, raising concerns about safety and long-term viability. Third, the timing of intervention is a critical but often overlooked factor. Early-stage blockades of cytokines may prevent BBB disruption but later-stage interventions may be less effective or even detrimental, particularly in chronic conditions.

Moreover, animal models, especially rodents, exhibit substantial differences from humans in terms of BBB structure and function, limiting the direct extrapolation of findings. Preclinical studies often utilize acute experimental setups that fail to replicate the chronic and progressive nature of BBB dysfunction observed in clinical conditions. Variability in cytokine administration methods, dosages, and exposure durations further complicate cross-study comparisons.

In vitro models, such as iPSC-derived BBB co-cultures, remain inadequate in capturing the complexity of the human BBB. Additionally, preclinical research frequently neglects comorbidities, such as diabetes or hypertension, which can significantly alter cytokine signaling and compromise BBB integrity. The absence of reliable biomarkers for detecting BBB disruption and cytokine-induced damage hampers both diagnostic efforts and therapeutic monitoring.

Despite promising preclinical findings, clinical trials targeting cytokine pathways have often yielded inconsistent outcomes due to differences in drug delivery methods, pharmacokinetics, and patient heterogeneity. Furthermore, therapeutic strategies focused solely on cytokine inhibition may overlook interactions with other signaling pathways essential for maintaining BBB integrity. Addressing these limitations will require more accurate models, the incorporation of relevant comorbid conditions, and the development of biomarkers that reliably reflect BBB dysfunction in clinical contexts.

In conclusion, while understanding the mechanisms by which proinflammatory cytokines affect the BBB is essential, translating these insights into effective therapies remains a challenge. Continued research is needed to bridge the gap between preclinical successes and clinical efficacy, particularly in developing drugs capable of penetrating the BBB and addressing the complex, multifactorial nature of BBB dysfunction. Such efforts are crucial for advancing treatment strategies for pathological changes in nervous tissue, including brain tumors and other neuroinflammatory conditions. 

## Figures and Tables

**Table 1:** Influence of proinflammatory cytokines and chemokines on the blood–brain barrier (BBB) structure and function.

Molecule	Category of Action	Impact on BBB (Structures)	Clinical Relevance (Pathology, Therapy)	References
IL-1	Proinflammatory	Disrupts tight junctions of capillary endothelial cells; activates other proinflammatory cytokines; abolishes the protective effect of astrocytes on BBB integrity by suppressing astrocytic sonic hedgehog (SHH) production; stimulates astrocytes to produce potential neurotoxic substances; stimulates vascular permeability and angiogenesis	Involvement in neuroinflammation; potential therapeutic target	[94,95,96,97,98,99,100]
IL-1β	Proinflammatory	Disrupts tight junctions of capillary endothelial cells; damages astrocytes by downregulating SHH; increases the secretion of other proinflammatory cytokines	Involvement in neuroinflammation; potential therapeutic target	[97,98,101,102]
TNF-α	Proinflammatory	Damages tight junctions; induces astrocyte dysfunction; alters BBB morphology	Pathogenesis of MS, NMOSD, depressive states; therapeutic target	[103,104,105,106,107]
IFN-γ	Proinflammatory	Disrupts tight junctions of endothelial cells; induces transendothelial migration of CD4+ T cells to the basement membrane and promotes the transcellular route of this migration; induces a change in the C6 of ZO-1 and decreases mRNA and protein levels of ZO-1 in epithelial cells	Autoimmune CNS diseases; potential therapeutic target	[108,109,110,111]
IL-17A	Proinflammatory	Disrupts the tight junctions of endothelial cells by downregulating the expression of occludin; enhances neuroinflammatory signaling pathways	Migraine, MS, neuropsychiatric lupus; promising therapeutic target	[112]
IL-6	Mixed	Disrupts tight junctions of capillary endothelial cells; reduces tight junction proteins β-catenin; may act anti-inflammatory by reducing the secretion of other proinflammatory cytokines	NMOSD, MS, Alzheimer’s; potential therapeutic target	[11,90,113,114,115,116]
IL-2	Mixed	Activates endothelium, may increase BBB permeability; supports BBB repair in certain conditions; astrocyte-targeted IL-2 gene delivery may protect against neuroinflammation and BBB disruption by an increase in brain resident regulatory T cell number	MS, Treg-based therapies; potential therapeutic target	[117,118,119,120]
IL-12	Mixed	Promotes neuroinflammation and BBB disruption; in autoimmune neuroinflammation in mice, plays a neuroprotective role that is mediated by neuroectoderm-derived cells, specifically neurons, and not immune cells	MS, anti-IL-12/23 therapies	[121,122,123]
IL-15	Mixed	Has a low level of permeability through the BBB; reduces astrocyte damage and death, increasing resistance to cytotoxicity; IL-15 complex treatment during experimental cerebral malaria in mice reduces BBB permeability and prevents BBB breakdown (the effect related to the induction of IL-10-producing NK cells)	HIV, MS, potential therapeutic application	[124,125,126,127]
IL-18	Mixed	Mediated up-regulation of dystrophin expression may play either a direct or indirect role in the maintenance of BBB function following status epilepticus (prevents increased BBB permeability leading to vasogenic edema)	Inflammatory states in epilepsy, MS; potential therapeutic target	[128]
IL-4	Anti-inflammatory	May influence BBB permeability through modulation of the immune response and interaction with microglial cells	Elevated IL-4 levels in serum are associated with markers of BBB damage in paraneoplastic syndromes; neutralization of IL-4 may protect dopaminergic neurons and reduce BBB damage in Parkinson’s disease models	[129,130,131]
CCL2 (MCP-1—Monocyte Chemoattractant Protein-1)	Proinflammatory	Attracts monocytes to the BBB, facilitates transmigration	MS, neuroinfections, stroke; therapeutic target	[97]
CXCL12 (SDF-1—Stromal Cell-Derived Factor 1)	Proinflammatory	Mobilization of progenitor cells, angiogenesis, regulation of migration of stem cells and lymphocytes	Cancer metastasis, immune responses	[132]
CCL20 (MIP-3α—Macrophage Inflammatory Protein-3 alpha)	Proinflammatory	Facilitates Th17 cell migration across the BBB	MS, therapeutic target	[132]
CX3CL1 (Fractalkine)	Proinflammatory	Attracts neutrophils, damages endothelial cells	Meningitis, stroke; potential therapeutic target	[133,134,135,136,137]
Histamine	Proinflammatory	Vasodilation, increased vascular permeability, recruitment of inflammatory cells	Allergic reactions, inflammation, regulation of immune response	[138,139,140]

*Abbreviations:* IFN-γ—interferon gamma; IL-1—interleukin 1; IL-2—interleukin 2; IL-6—interleukin 6; IL-12—interleukin 12; IL-15—interleukin 15; IL-17—interleukin 17; IL-18—interleukin 18; TNF-α—tumor necrosis factor alpha; ZO-1—zonula occludens-1. *Source:* compiled by the authors.

## Data Availability

No new data were created or analyzed in this study. Data sharing is not applicable to this article.

## Abbreviations

BBB—blood–brain barrier; BMECs—brain microvascular endothelial cells; CCL2—C-C motif chemokine ligand 2, also known as monocyte chemotactic protein 1 (MCP1); CCL19—C-C motif chemokine ligand 19, also known as macrophage inflammatory protein-3 beta (MIP-3β); CCL20—C-C motif chemokine ligand 20; CCL21—C-C motif chemokine ligand 21; CD13—cluster of differentiation 13, also known as aminopeptidase N (APN) or alanyl aminopeptidase; CNS—central nervous system; COVID-19—coronavirus disease 2019; CSF—cerebrospinal fluid; CVOs—circumventricular organs; CX3CL1—C-X3-C motif chemokine ligand 1, also known as fractalkine; CXCL12—C-X-C motif chemokine ligand 12, also known as stromal cell-derived factor 1 (SDF-1); e-*τ*—extracellular tau aggregates; GLUT1—glucose transporter 1; HDC—histidine decarboxylase; HSPG—heparan sulfate proteoglycan; ICAM-1—intercellular adhesion molecule-1, also known as cluster of differentiation 54 (CD54); IFN-γ—Interferon gamma; IL-1, IL-1α, IL-1β, IL-2, IL-4, IL-6, IL-10, IL-12, IL-15, IL-17, IL-18—interleukins: interleukin 1, interleukin 1-alpha, interleukin 1-beta, interleukin 2, interleukin 4, interleukin 6; interleukin 10, interleukin 12, interleukin 15, interleukin 17, interleukin 18, respectively; JAMs—junctional adhesion molecules; kDa—kilodalton (a non-SI unit of mass); MAPT—microtubule-associated protein tau; MMP-2, MMP-3, MMP-9—matrix metalloproteinases 2, 3 and 9, respectively; NGR-TNF—a drug created by fusing a peptide Cys-Asn-Gly-Arg-Cys-Gly (CNGRCG), denoted as NGR, with a TNF-α molecule; NK cells—natural killer cells; NMO-IgG—neuromyelitis optica immunoglobulin G; PCs—pericytes; P/E selectins—platelet/endothelial selectins, also known as CD62P/CD62E, respectively; RNAs—ribonucleic acids; ROS—reactive oxygen species; S100B—S100 calcium-binding protein beta; SARS-CoV-2—severe acute respiratory syndrome coronavirus 2; SHH—sonic hedgehog homolog (protein); SIT—saturated influx transport; TBI—traumatic brain injury; TIMP-1, TIMP-2—tissue inhibitors of metalloproteinases 1 and 2, respectively; TNF-α—tumor necrosis factor alpha; TSP-2—thrombospondin-2; VCAM-1—vascular cell adhesion molecule 1, also known as cluster of differentiation 106 (CD106); VEGF—vascular endothelial growth factor; ZO-1, ZO-2 and ZO-3—zonula occludens proteins 1, 2, and 3, respectively.
